# Warmer weather unlikely to reduce the COVID-19 transmission: An ecological study in 202 locations in 8 countries

**DOI:** 10.1016/j.scitotenv.2020.142272

**Published:** 2021-01-20

**Authors:** Jinhua Pan, Ye Yao, Zhixi Liu, Xia Meng, John S. Ji, Yang Qiu, Weidong Wang, Lina Zhang, Weibing Wang, Haidong Kan

**Affiliations:** a School of Public Health, Key Lab of Public Health Safety of the Ministry of Education, Fudan University, Shanghai 200032, China; b Environmental Research Center, Duke Kunshan University, Kunshan, Jiangsu, China; c Department of Environmental Sciences and Engineering, School of Architecture and Environmental Sciences, Sichuan University, Chengdu, China; d Shanghai Key Laboratory of Meteorology and Health, Shanghai, China; e Nicholas School of the Environment, Duke University, Durham, NC, USA

**Keywords:** SARS-CoV-2, Meteorological factors, Temperature, Ultraviolet radiation

## Abstract

**Purpose:**

To examine the association between meteorological factors (temperature, relative humidity, wind speed, and UV radiation) and transmission capacity of COVID-19.

**Methods:**

We collected daily numbers of COVID-19 cases in 202 locations in 8 countries. We matched meteorological data from the NOAA National Centers for Environmental Information. We used a time-frequency approach to examine the possible association between meteorological conditions and basic reproductive number (R_0_) of COVID-19. We determined the correlations between meteorological factors and R_0_ of COVID-19 using multiple linear regression models and meta-analysis. We further validated our results using a susceptible-exposed-infectious-recovered (SEIR) metapopulation model to simulate the changes of daily cases of COVID-19 in China under different temperatures and relative humidity conditions.

**Principal results:**

Temperature did not exhibit significant association with R_0_ of COVID-19 (meta *p* = 0.446). Also, relative humidity (meta *p* = 0.215), wind speed (meta *p* = 0.986), and ultraviolet (UV) radiation (meta *p* = 0.491) were not significantly associated with R_0_ either. The SEIR model in China showed that with a wide range of meteorological conditions, the number of COVID-19 confirmed cases would not change substantially.

**Conclusions:**

Meteorological conditions did not have statistically significant associations with the R_0_ of COVID-19. Warmer weather alone seems unlikely to reduce the COVID-19 transmission.

## Highlights

• Temperature did not exhibit significant associations with COVID-19's R_0_.
• Other meteorological factors weren't significantly associated with COVID-19's R_0_.
• It's unlikely to depend on warmer weather to reduce the COVID-19 transmission.

## Graphical abstract

**Figure:**
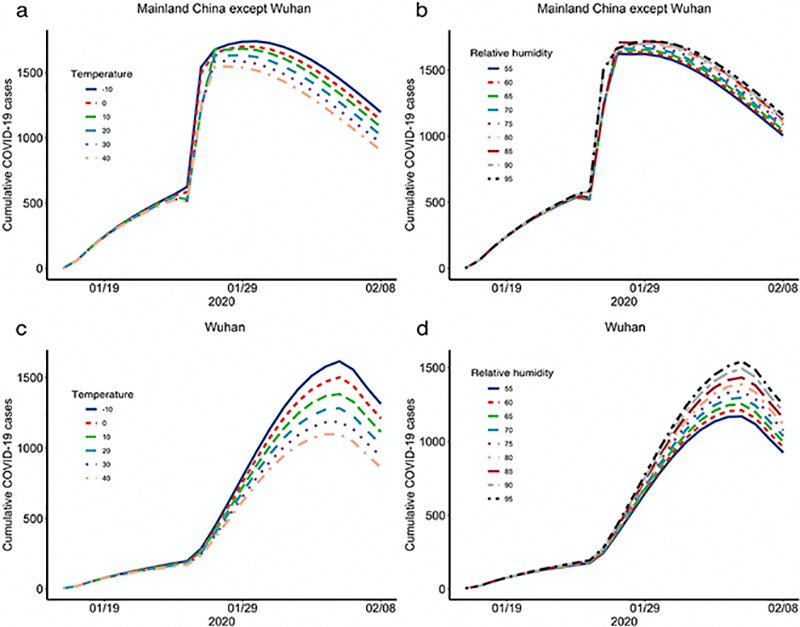

## 1 Introduction

The novel coronavirus disease 2019 (COVID-19) is an infectious disease caused by severe acute respiratory syndrome coronavirus 2 (SARS-CoV-2). It was first identified in December of 2019 in Wuhan, China, and has since become an ongoing pandemic. It has impeded global development and stressed health care delivery systems worldwide (Li et al., 2020). The number of confirmed COVID-19 cases worldwide is still increasing; as of Aug 31, 2020, more than 200 countries reported a total of 25,302,607 confirmed cases. The infectious capacity of SARS-CoV-2 is higher than Middle East Respiratory Syndrome Coronavirus (MERS-CoV), and severe acute respiratory syndrome (SARS), which is a viral respiratory disease of zoonotic origin caused by severe acute respiratory syndrome coronavirus (SARS-CoV or SARS-CoV-1) (Meo et al., 2020).

Previous studies have shown the importance of meteorological conditions in the transmission of infectious diseases, including, but not limited to, influenza and severe acute respiratory syndrome (SARS). For example, it was reported that the transmission of SARS can be influenced by meteorological factors (*e*.*g*., temperature, relative humidity) (Xu et al., n.d.). Given the similarities between SARS virus and SARS-CoV-2, many researchers hypothesize that temperature, relative humidity, and UV radiation could play a similar role in the COVID-19 transmission (Xu et al., n.d.). Several earlier studies have investigated the association between temperature, relative humidity, UV radiation, and COVID-19; however, these studies reached different conclusions. Yao et al. analyzed the association between temperature, relative humidity, and UV radiation with COVID-19 transmission rate in 62 Chinese cities and reported that warmer temperature could not mitigate the epidemic; besides, the relative humidity and UV radiation had no relationship with COVID-19 transmission in China (Yao et al., 2020). The findings from studies in Spain and Iran were also consistent with the Chinese study (Briz-Redon and Serrano-Aroca, 2020; Sahafizadeh and Sartoli, 2020). Other studies, however, came to an opposite conclusion that meteorological factors, such as temperature and relative humidity, were associated with confirmed COVID-19 cases (Byass, 2020; Pani et al., 2020; Runkle et al., 2020). The different conclusions came from these aforementioned studies may partially contributed to the different statistical and modelling approaches or due to their limited generalizability globally as they were conducted within one particular country with meteorological conditions specific to the local climate.

Therefore, this study aims to comprehensively examine the associations between meteorological factors (temperature, relative humidity, and wind speed) and COVID-19 transmission at a global scale. Further, we validated our results using a susceptible-exposed-infectious-recovered (SEIR) metapopulation model to simulate changes of daily cases of COVID-19 in China under different temperatures and relative humidity conditions.

## 2 Material and methods

### 2.1 Health data

From the databases compiled by Johns Hopkins University, we collected data on the daily reported COVID-19 cases in 7 locations in Australia, 9 locations in Canada, and 50 locations in the United States (US). From the respective Ministries of Health, we collected COVID-19 data in 63 locations in China (2020c), 8 locations in Germany (2020e), 19 locations in Italy, 5 locations in Japan (2020b), and 41 locations in the United Kingdom (UK) (2020a).

All these locations have cumulative number of cases over 50 to enable estimation of the basic reproductive number (R_0_) of COVID-19. In brief, R_0_ represents the expected number of secondary cases produced by an initial infectious individual in a completely susceptible population (Pan et al., 2020). In order to obtain stable R_0_ and to avoid influences of difference in detection methods, inspection strategies and reported ways on the number of confirmed cases in different countries, we determined the initial days for various locations according to the following criteria: 1) there should be consecutive case days lasting at least four days, and 2) the number of newly confirmed cases should not equal to one in any of these four days, then 3) the first day of the aforementioned dates was chosen as the first case-day in the study regions. In each location, a total of 18 days (17 days after of the initial date and including the initial date) were used to estimate R_0_.

We employed the method introduced by King et al. to estimate R_0_ (King et al., 2017). First, we constructed a linear regression model to estimate the correlation coefficients. Second, we obtained R_0_ by combining the coefficients obtained derived from the previous step with the average incubation and confirmation periods. We assigned the average values of the incubation period and the average days from infection to confirmation as 7.0 and 3.8 days, respectively, according to a previous research (Li et al., 2020).

### 2.2 Meteorological data

Meteorological parameters include temperature, relative humidity, wind speed, and UV radiation. We collected hourly data of temperature, relative humidity, and wind speed from the NOAA National Centers for Environmental Information (2020d). Daily mean levels of these meteorological factors were calculated at station level and then aggregated to location levels to be matched with R_0_ of COVID-19.

Regarding UV radiation, daily erythemally weighted daily dose (EDD) data were extracted from the Dutch-Finnish Ozone Monitoring Instrument (OMI) Level 2 UV irradiance products with version 003 (OMUVB V003) at 13 km × 24 km resolution. OMI is a nadir-viewing spectrometer aboard the NASA Aura satellite covering UV wavelength from 270 to 380 nm. The average of EDD values from OMI pixels matched within these locations was assigned as the daily mean EDD level for the corresponding locations.

### 2.3 Statistical analysis

Firstly, we used the wavelet coherence analysis to examine the possible association between meteorological factors and COVID-19 cases in 198 locations. Abruzzo in Italy, Stockton-on-Tees and Suffolk of the UK, and Delaware of the US were excluded from this analysis because of missing data in daily temperature for some days. The association between daily meteorological factors and numbers of COVID-19 cases in these locations was measured by the mathematical “magnifying glass” of wavelet coherency analysis (He et al., 2018). The method quantitatively represents the internal covariates between the two-time series according to the synchronization intensity of the two time series trends. In the present analysis, the daily time series pairs (*e*.*g*. COVID-19 cases and temperature) were used to implement wavelet coherency analysis (He et al., 2018). Larger coherency value implies stronger association.

Secondly, we estimated the country-specific associations between meteorological factors and R_0_ of COVID-19 in the same period using multiple regression models. We then used meta-analysis to pool the *p* values of the country-specific associations of meteorological factors with R_0_.

Finally, to validate the observed associations of meteorological factors with the COVID-19 transmission, we constructed a susceptible-exposed-infectious-recovered (SEIR) model (Fig. S1) using Chinese data. We chose China because more detailed meteorological data and case information were available only in the Chinese cities. Specifically, when a susceptible (S) person encounters a COVID-19 case, this person is likely to be infected by the virus (E) and progress to the infected phase (I), and the person may appear to have obvious symptoms and be subsequently diagnosed. The patients might be treated (T), or they have been showing asymptomatic infection and stay in sub-clinical stage (U), and then most of them will join the rehabilitation group (recovery, R). The flow diagram for the model appears in Fig. S1 and the formula for the model are shown below, of which β_1_ represents the probability of transmission following an effective contact between infectious and exposed cases and susceptible individuals, β_2_ represents the probability of transmission following a contact between subclinical cases and susceptible individuals, q is the quarantine rate, j is the detection rate, α_1_ is the death rate, γ_1_ and d are progression rate of cases from confirmed to recovery and exposed to infection, respectively. μ_1_ and μ_2_ are ratios of subclinical and confirmed cases. We constructed the SEIR models for Wuhan and other Chinese cities separately, given the dominant COVID-19 cases from Wuhan. In the SEIR models, we estimated the R_0_ under various temperature and relative humidity using the relationship established in the regression models. We then estimated daily number of confirmed cases and new cases under expanded range of temperature and humidity (the range of temperature: −10 to 40 degrees; the range of relative humidity: 55% to 95%). Then we used one-way ANOVA analysis to test the differences between the daily confirmed cases of COVID-19 under different meteorological conditions.

The detailed formula in the SEIR model are as the following:

$$\frac{\mathrm{dS}}{\mathrm{dt}}=-\mathrm{Sq}-\beta_{1}S\left(I+E\right)-\beta_{2}\mathrm{SU}$$

$$\frac{\mathrm{dE}}{\mathrm{dt}}=\beta_{1}S\left(I+E\right)+\beta_{2}\mathrm{SU}-\mathrm{Eq}-\mathrm{dE}$$

$$\frac{\mathrm{dI}}{\mathrm{dt}}=\mathrm{dE}-\mu_{1}I-{\mathrm{j\mu}}_{2}I-\alpha_{1}I$$

$$\frac{\mathrm{dU}}{\mathrm{dt}}=\mu_{1}I-\gamma_{2}U-\alpha_{1}U$$

$$\frac{\mathrm{dT}}{\mathrm{dt}}={\mathrm{j\mu}}_{2}I-\gamma_{1}T-\alpha_{1}T$$

$$\frac{\mathrm{dR}}{\mathrm{dt}}=\gamma_{2}U+\gamma_{1}T$$

All calculations were completed in R software version 3.6.1 (R Foundation for Statistical Computing) and MATLAB R2019b. A *p* value of less than 0.05 was considered to indicate statistical significance.

## 3 Results

### 3.1 Descriptive characteristics

Among the 202 locations, the mean ± standard deviation and range of R_0_ of COVID-19 were (1.7 ± 0.5, 0.6–4.0) in 202 districts. The top three locations with highest R_0_ were Hamburg and Hessen in Germany and New York in the US. The average temperature was 6.7 °C, with a range of −23.0 °C to 29.6 °C and the median ± interquartile range for temperature in these locations were shown in Supplemental Table 1.

**Fig. 1:**
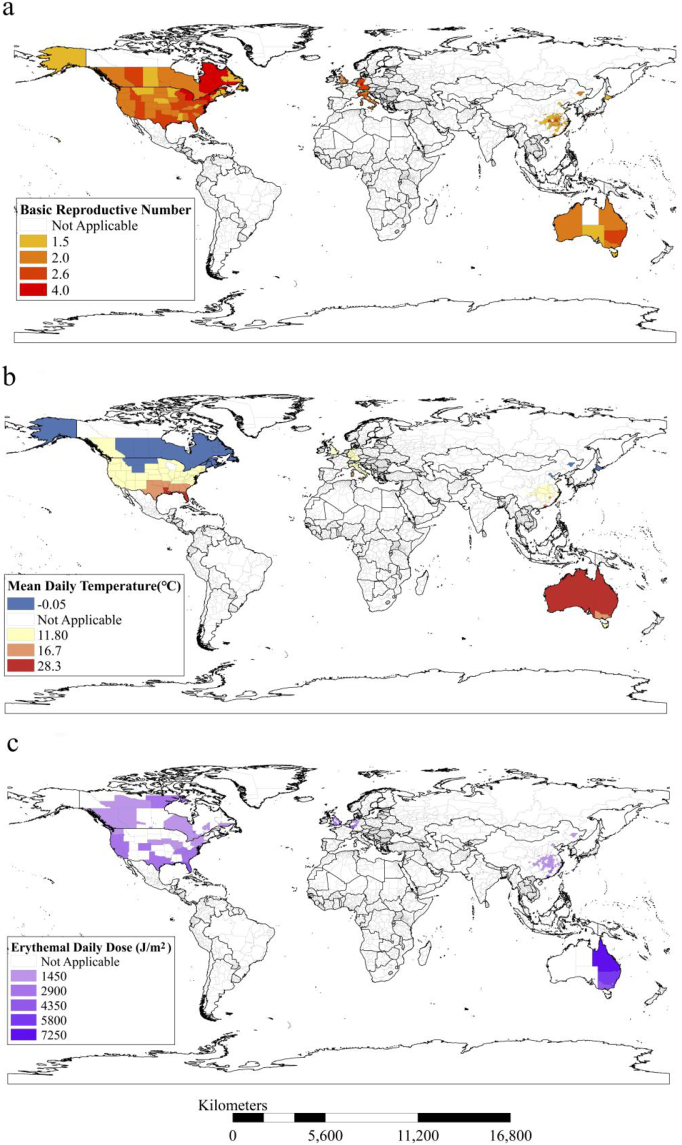
The spatial distribution of temperature and R_0_ of COVID-19 in the 202 districts. Panel a exhibited the geographic distribution of basic reproductive number of the 202 districts in the North America, Europe, Oceania, and Asia. The R_0_ values exhibited in the map are the R_0_ values of 18 days calculated for each administrative region. Panel b showed the geographic distribution of the 202 districts in the North America, Europe, Oceania, and Asia that had available data on temperature which is included in the analysis. The temperature data are shown as the mean value. Panel c showed the geographic distribution of the 134 districts in the North America, Europe, Oceania, and Asia that had available data on UV radiation data.

The meteorological conditions and R_0_ of COVID-19 in these locations exhibited different spatial patterns (Fig. 1). Generally, the locations far away from the equator have lower temperatures. The UV radiation tended to decrease with decreasing temperature (Fig. 1).

**Fig. 2:**
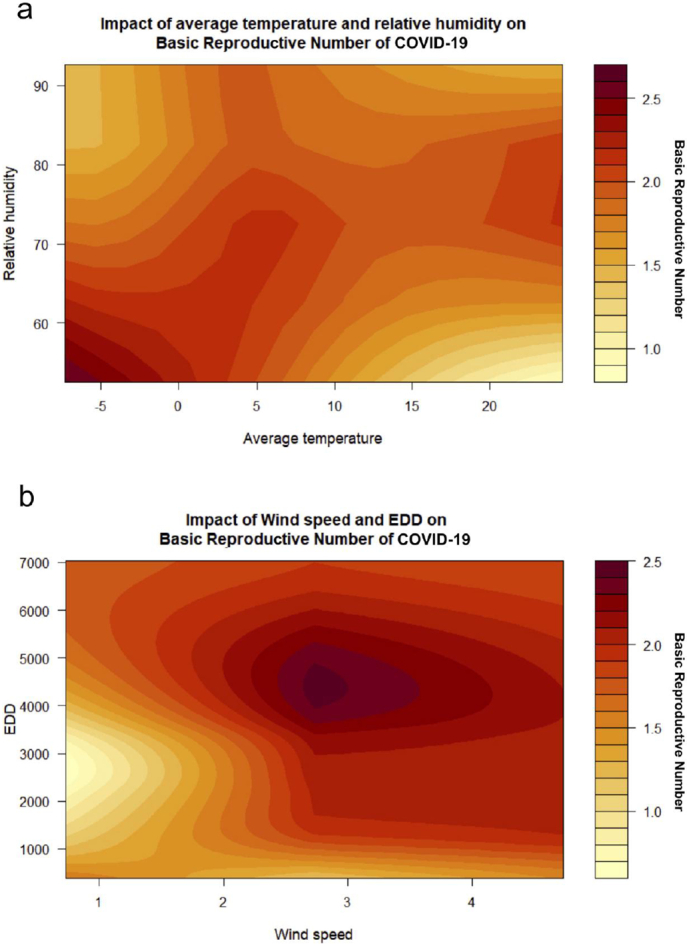
The relationship between meteorological factors and R_0_ of COVID-19. a: Association of average temperature, relative humidity, and basic reproductive number. The X- axis represents average temperature, Y-axis represents relative humidity, and the *Z*-axis represents R_0_. b: Association of wind speed, erythemal daily dose (EDD), and R_0_. The X-axis represents wind speed, Y-axis represents EDD, and the Z-axis represents R_0_.

When holding temperature constant, increasing relative humidity did not show uniform upward or downward trend in relation to R_0_ (Fig. 2a). There was no significant association between R_0_ and other meteorological parameters, such as wind speed or EDD (Fig. 2b).

### 3.2 Wavelet coherency analysis

**Fig. 3:**
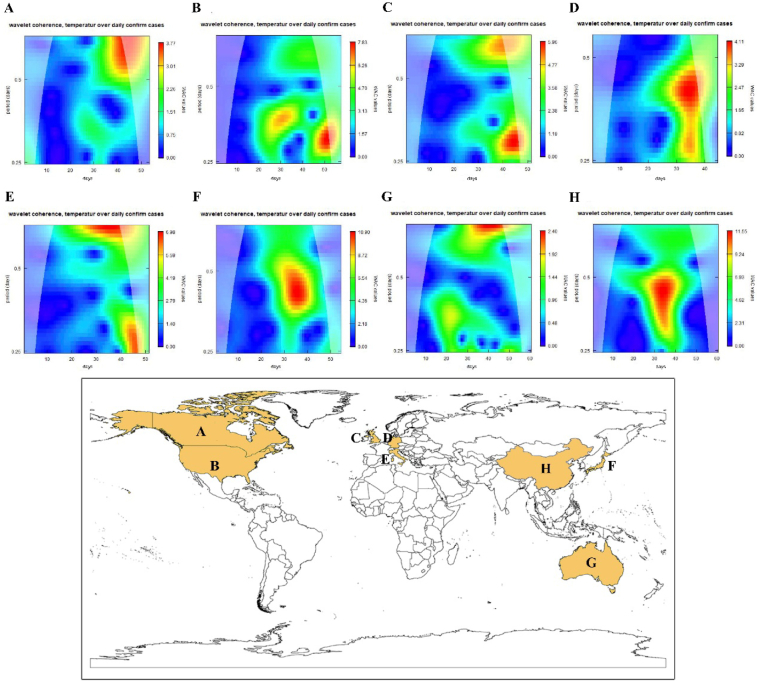
Global statistics of wavelet coherency spectra at all countries considered. Pane3 A~G are wavelet coherency spectra of different countries showing in the following map panel.

In total, 198 wavelet coherency spectra were obtained (one for each study region) (Fig. S2). Fig. 3 indicated that countries closer together showed more similar wavelet coherence figures. In general, we found that in these countries, the wavelet coherence value was relatively small, and only in a few days a relatively large wavelet coherence value was observed, suggesting temperature be less likely to be associated with COVID-19.

### 3.3 Meteorological factors and R_0_ of COVID-19

**Fig. 4:**
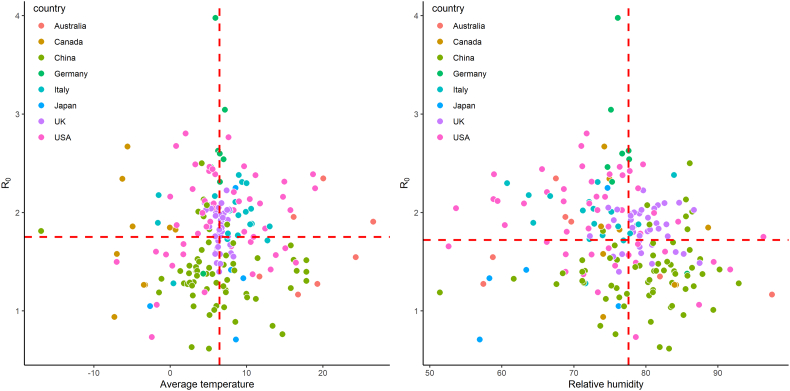
The distribution of temperature and relative humidity and R_0_ of COVID-19 in the studied locations. Fig. 4 shows the distribution of temperature and relative humidity and R_0_ of COVID-19 in the districts from different countries, and each color represent a country. The horizontal red line is the median of R_0_ and the vertical red line is the median of average temperature and relative humidity.

In the single-variable model, temperature exhibited no significant associations with R_0_ of COVID-19 (meta *p* = 0.446, see Fig. 4), showing that the COVID-19 transmission would not change with increasing temperature.

**Fig. 5:**
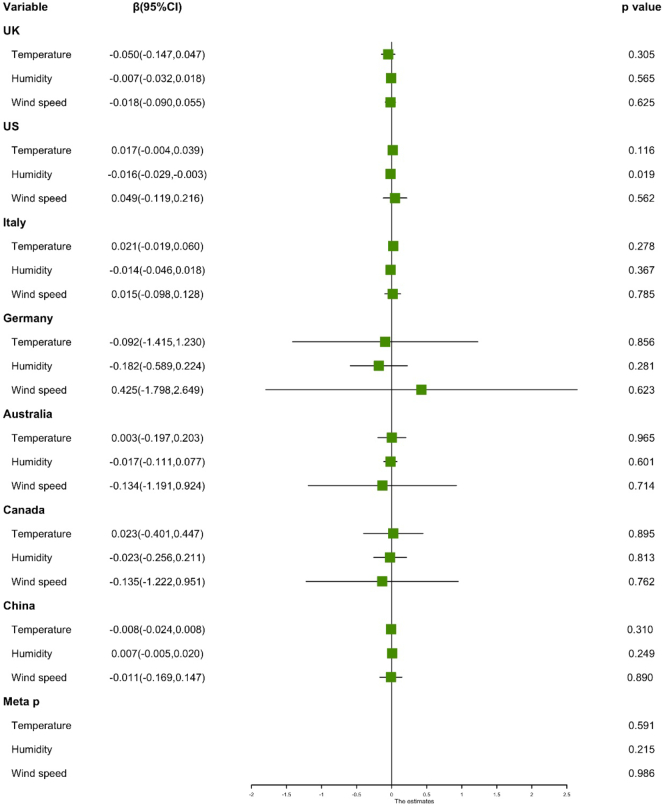
The relationship between other meteorological factors and R_0_ of COVID-19. Results of multiple linear regression models are represented as estimate and 95% confidence intervals. Student *t*-tests were performed to calculate the *p*-values. The green square represents the value of estimate. The left and right lines of the green square represent the range of the confidence interval. The black line represents the baseline of estimate.

In the 200 locations with complete meteorological factors, multi-variable regression analysis found that temperature (meta *p* = 0.591), relative humidity (meta *p* = 0.215), and wind speed (meta *p* = 0.986) were not significantly associated with R_0_ (Fig. 5), suggesting that the transmission capacity of COVID-19 would not change with the variation of temperature, wind speed, or relative humidity.

**Fig. 6:**
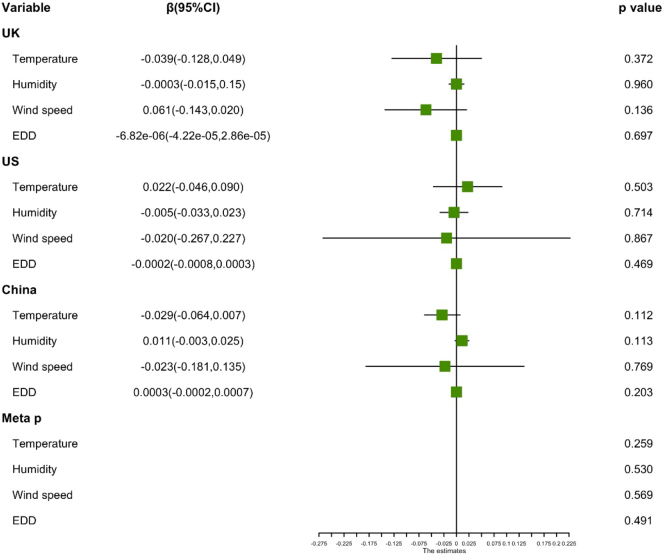
The relationship between UV and R_0_ of COVID-19. Results of multiple linear regression models are represented as estimate and 95% confidence intervals. Student *t*-tests were performed to calculate the p-values. The green square represents the value of estimate. The left and right lines of the green square represent the range of the confidence interval. The black line represents the baseline of estimate.

The UV radiation data were collected only in 134 locations in China, UK and US. After adjustment for temperature, wind speed, and relative humidity, there was no significant association between UV and R_0_ (meta *p* = 0.491) either (Fig. 6).

### 3.4 SEIR model in China

**Fig. 7:**
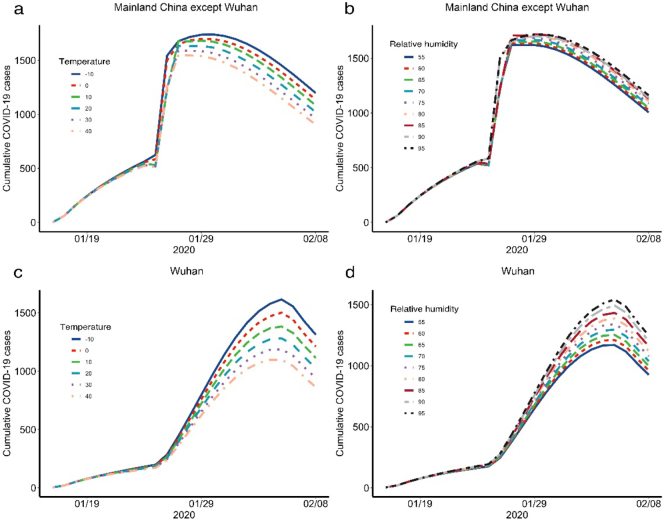
The changes of daily confirm cases of COVID-19 under different daily temperature and relative humidity in China. Panel a and panel c are the changes of daily confirm cases of COVID-19 under different daily temperature in mainland China except Wuhan and in Wuhan, panel b and panel d are the changes of daily confirm cases of COVID-19 under different relative humidity in mainland China except Wuhan and in Wuhan.

Fig. 7 shows the change of daily new confirmed cases in China under different meteorological conditions. When temperature increases, daily confirmed cases of COVID-19 would not change significantly in Wuhan (F = 0.467, *p* = 0.800) or other Chinese cities (F = 0.241, *p* = 0.944). Similarly, we did not find significant changes in the number of COVID-19 cases associated with decreasing relative humidity in Wuhan (F = 0.056, *p* = 1.000) and other Chinese cities (F = 0.201, *p* = 0.990).

## 4 Discussion

In this analysis covering 202 locations in 8 countries, meteorological conditions (temperature, wind speed, relative humidity, and UV radiation) were not significantly associated with the COVID-19 transmission, suggesting that warmer weather alone seems unlikely to reduce the spread ability of the pandemic. To our knowledge, this is the first study at a global scale to examine the relationship between meteorological conditions and epidemiological characteristics of COVID-19 using multiple regression models, mete-analysis, wavelet analysis and SEIR models.

Previous studies reported that certain meteorological factors may have effect on the transmission of respiratory-borne infectious diseases (Chan et al., 2011; Jaakkola et al., 2014). The conditions of successful transmission of respiratory pathogenic microorganisms are to maintain a certain degree of virulence in the whole airborne transmission process. The possible drivers may include temperature, humidity, UV radiation, and air ventilation (Duguid, 1946; Herfst et al., 2017). For example, Steel et al. demonstrated that the 2009 pandemic H1N1 influenza virus exhibited sensitivity to temperature and humidity, which was also characteristic of an H3N2 seasonal strain (Steel et al., 2011). Furthermore, Lowen et al. provided evidence that humidity and temperature conditions could affect the effective transmission of influenza viruses (Lowen et al., 2007). Besides, UV radiation is a major inactivating factor for influenza viruses in the outdoor environment (Weber and Stilianakis, 2008) and thus a high level of UV exposure may also constrain the transmission of SARS-COV virus (Rabenau et al., 2005). Therefore, it is hypothesized that COVID-19 transmission may decrease or even disappear when the temperature increases in the summer.

In this multi-country analysis, we did not observe the aforementioned relationship between temperature and transmission of COVID-19, even after adjusting for other potential confounders. Several prior studies also found that temperature did not play significant role in COVID-19 infection (Baker et al., 2020; Briz-Redon and Serrano-Aroca, 2020; Sahafizadeh and Sartoli, 2020; Yao et al., 2020), for example, Baker et al. used a climate-dependent epidemic model to simulate the COVID-19 pandemic, and found that without effective control measures, summer weather would not substantially limit pandemic growth (Baker et al., 2020). Briz-Redon et al. used spatial-temporal analysis to explore the relationship between cumulative number of COVID-19 cases and temperature, and found no evidence suggesting a reduction in COVID-19 cases at higher temperature (Briz-Redon and Serrano-Aroca, 2020). A few others reported opposite findings (Mendez-Arriaga, 2020; Qi et al., 2020; Shi et al., 2020; Tosepu et al., 2020). For example, Auler et al. (Auler et al., 2020). explored the relationship between meteorological conditions and the spread ability of COVID-19 in the most affected Brazilian cities and found higher mean temperatures and average relative humidity favored the COVID-19 transmission. Additionally, Shahzad et al. (2020) and Xie and Zhu (2020) also found a relationship between temperature and daily COVID-19 confirmed cases. To further verify our findings, we constructed a SEIR model using Chinese data and found that with increasing temperature, the new confirmed cases of COVID-19 did not decrease significantly. In this aspect, it might be premature to count on warm weather to stop the COVID-19 transmission.

Our study has strengths and limitations. The major strength is utilizing data from 202 locations in 8 countries, covering wide climate areas and enabling our findings generalized globally. Also, there are several limitations of this study. Firstly, our major outcome, R_0_ of COVID-19, is influenced by a number of factors such as various lockdown policies across countries, different phases of the COVID-19 epidemic, as well as other unmeasured confounders. In response, we conducted a two-stage analysis (first within country, and then multi-country) to account for the variation of control policy across countries. Also, to facilitate a comparison of R_0_ across various locations, we uniformly chose a total of 18 days in each location (17 days after of the initial date and including the initial date) to calculate R_0_. Secondly, our study is ecological in nature, with lack of individual-level (*e*.*g*. patients' age, sex) and some location-specific information (*e*.*g*. intensity of control policy, availability of medical resources), which may have an influence on the COVID-19 transmission and confound our results. Another limitation is the use of relative humidity, which is be highly related to temperature and may is not an adequate measure of humidity. Also, we chose these 202 locations because detailed daily data of new confirmed COVID-19 cases and environmental factors of these locations are available in the public database, which may cause selection bias to some extent. Future studies should develop complicated models with higher spatial-temporal resolution to assess the relationship between meteorological conditions and the epidemiological characteristics of COVID-19.

## 5 Conclusions

Conclusively, this study provides the first global evidence that meteorological factors do not have significant effect on the COVID-19 transmission. Given the lack of association, public health agencies should not rely on the warm weather to flatten the curve of COVID-19 transmission. Therefore, the non-medication interventions should be implemented consistently to keep constraining the epidemic caused by SARS-CoV-2, in case of another resurgence.

## Funding

This work was supported by the 10.13039/100000865Bill & Melinda Gates Foundation, Seattle, WA [Grant No. OPP1216424] and the Fudan University Research Project on COVID-19 Emergency, China, SH [Grant No. IDF201007].

## Role of the funding source

The funders of the study had no role in study design, data collection, data analysis, data interpretation, or writing of the report. The corresponding author had full access to all the data in the study and had final responsibility for the decision to submit for publication.

## Ethical approval

The Institutional Review Board at the School of Public Health, Fudan University, approved the study protocol with a waiver of informed consent. Data were analyzed at aggregate level and no participants were contacted.

## CRediT authorship contribution statement

**Jinhua Pan**: Investigation, Methodology, Formal analysis and Roles/Writing-original draft. **Ye Yao**: Methodology, Formal analysis, Software and Writing-Review and Editing. **Xia Meng**: Investigation, Data curation, Visualization and Writing-Review and Editing. **Zhixi Liu**: Investigation, Software, Visualization and Roles/Writing-original draft. **John S Ji**: Investigation, Writing-Review and Editing. **Yang Qiu**: Data curation, Writing-Review and Editing. **Weidong Wang**: Investigation, Data curation. **Lina Zhang**: Investigation, Data curation. **Weibing Wang**: Conceptualization, supervision, funding acquisition and writing-review and editing. **Haidong Kan**: Conceptualization, supervision, resources and Writing-review and editing. All authors critically reviewed and approved the final version of the manuscript. The corresponding authors are responsible for ensuring that the descriptions are accurate and agreed by all authors.

## Declaration of competing interest

The authors declare that they have no known competing financial interests or personal relationships that could have appeared to influence the work reported in this paper.

## Appendix A Supplementary data

Supplementary material Image 1

## References

[bb0005] Auler A.C., Cassaro F.A.M., da Silva V.O., Pires L.F. (2020). Evidence that high temperatures and intermediate relative humidity might favor the spread of COVID-19 in tropical climate: a case study for the most affected Brazilian cities. Sci. Total Environ..

[bb0010] Baker R.E., Yang W., Vecchi G.A., Metcalf C.J.E., Grenfell B.T. (2020). Susceptible supply limits the role of climate in the early SARS-CoV-2 pandemic. Science.

[bb0025] Briz-Redon A., Serrano-Aroca A. (2020). A spatio-temporal analysis for exploring the effect of temperature on COVID-19 early evolution in Spain. Sci. Total Environ..

[bb0030] Byass P. (2020). Eco-epidemiological assessment of the COVID-19 epidemic in China, January-February 2020. Glob. Health Action.

[bb0035] Chan K.H., Peiris J.S., Lam S.Y., Poon L.L., Yuen K.Y., Seto W.H. (2011). The effects of temperature and relative humidity on the viability of the SARS coronavirus. Adv Virol.

[bb0040] Duguid J.P. (1946). The size and the duration of air-carriage of respiratory droplets and droplet-nuclei. J Hyg (Lond).

[bb0045] He J., Christakos G., Wu J., Cazelles B., Qian Q., Mu D. (2018). Spatiotemporal variation of the association between climate dynamics and HFRS outbreaks in Eastern China during 2005-2016 and its geographic determinants. PLoS Negl. Trop. Dis..

[bb0050] Herfst S., Bohringer M., Karo B., Lawrence P., Lewis N.S., Mina M.J. (2017). Drivers of airborne human-to-human pathogen transmission. Curr Opin Virol.

[bb0060] Jaakkola K., Saukkoriipi A., Jokelainen J., Juvonen R., Kauppila J., Vainio O. (2014). Decline in temperature and humidity increases the occurrence of influenza in cold climate. Environ. Health.

[bb0070] Johns Hopkins University COVID-19 data repository. https://github.com/CSSEGISandData/COVID-19.

[bb0075] King Aaron A., Bjørnstad Ottar, Bolker Ben, Drake John, Rohani Pej, Smith D. (2017). Introduction to Model Parameter Estimation.

[bb0080] Li Q., Guan X., Wu P., Wang X., Zhou L., Tong Y. (2020). Early transmission dynamics in Wuhan, China, of novel coronavirus-infected pneumonia. N. Engl. J. Med..

[bb0085] Lowen A.C., Mubareka S., Steel J., Palese P. (2007). Influenza virus transmission is dependent on relative humidity and temperature. PLoS Pathog..

[bb0090] Mendez-Arriaga F. (2020). The temperature and regional climate effects on communitarian COVID-19 contagion in Mexico throughout phase 1. Sci. Total Environ..

[bb0095] Meo S.A., Alhowikan A.M., Al-Khlaiwi T., Meo I.M., Halepoto D.M., Iqbal M. (2020). Novel coronavirus 2019-nCoV: prevalence, biological and clinical characteristics comparison with SARS-CoV and MERS-CoV. Eur. Rev. Med. Pharmacol. Sci..

[bb0105] NOAA National Centers for Environmental Information Integrated surface database. https://www.ncdc.noaa.gov/isd.

[bb0110] Pan J., Yao Y., Liu Z., Li M., Wang Y., Dong W. (2020). Effectiveness of Control Strategies for Coronavirus Disease 2019: A SEIR Dynamic Modeling Study.

[bb0115] Pani S.K., Lin N.H., RavindraBabu S. (2020). Association of COVID-19 pandemic with meteorological parameters over Singapore. Sci. Total Environ..

[bb0120] Qi H., Xiao S., Shi R., Ward M.P., Chen Y., Tu W. (2020). COVID-19 transmission in Mainland China is associated with temperature and humidity: a time-series analysis. Sci. Total Environ..

[bb0125] Rabenau H.F., Cinatl J., Morgenstern B., Bauer G., Preiser W., Doerr H.W. (2005). Stability and inactivation of SARS coronavirus. Med. Microbiol. Immunol..

[bb0135] Runkle J.D., Sugg M.M., Leeper R.D., Rao Y., Matthews J.L., Rennie J.J. (2020). Short-term effects of specific humidity and temperature on COVID-19 morbidity in select US cities. Sci. Total Environ..

[bb0140] Sahafizadeh E., Sartoli S. (2020). High Temperature Has no Impact on the Reproduction Number and New Cases of COVID-19 in Bushehr, Iran.

[bb0145] Shahzad F., Shahzad U., Fareed Z., Iqbal N., Hashmi S.H., Ahmad F. (2020). Asymmetric nexus between temperature and COVID-19 in the top ten affected provinces of China: a current application of quantile-on-quantile approach. Sci. Total Environ..

[bb0150] Shi P., Dong Y., Yan H., Zhao C., Li X., Liu W. (2020). Impact of temperature on the dynamics of the COVID-19 outbreak in China. Sci. Total Environ..

[bb0155] Steel J., Palese P., Lowen A.C. (2011). Transmission of a 2009 pandemic influenza virus shows a sensitivity to temperature and humidity similar to that of an H3N2 seasonal strain. J. Virol..

[bb0160] Tosepu R., Gunawan J., Effendy D.S., Ahmad O.A.I., Lestari H., Bahar H. (2020). Correlation between weather and Covid-19 pandemic in Jakarta, Indonesia. Sci. Total Environ..

[bb0165] Weber T.P., Stilianakis N.I. (2008). Inactivation of influenza a viruses in the environment and modes of transmission: a critical review. J. Inf. Secur..

[bb0170] Xie J., Zhu Y. (2020). Association between ambient temperature and COVID-19 infection in 122 cities from China. Sci. Total Environ..

[bb0175] Xu Ran, Rahmandad Hazhir, Gupta Marichi, DiGennaro Catherine, Ghaffarzadegan Navid, Amini Heresh The modest impact of weather and air pollution on COVID-19 transmission. https://projects.iq.harvard.edu/files/covid19/files/weather_and_covid-19_preprint.pdf.

[bb0180] Yao Y., Pan J., Liu Z., Meng X., Wang W., Kan H. (2020). No association of COVID-19 transmission with temperature or UV radiation in Chinese cities. Eur. Respir. J..

