# Supplementary material for: Warmer weather unlikely to reduce the COVID-19 transmission: An ecological study in 202 locations in 8 countries
Source: Sci Total Environ. 2021 Jan 20;753:142272. doi: 10.1016/j.scitotenv.2020.142272 (PMC7480263; doi:10.1016/j.scitotenv.2020.142272)
Supplement: Supplementary file 1 — Supplementary material [file mmc1.docx]

**Warmer weather unlikely to reduce the COVID-19 transmission: an ecological study in 202 locations in 8 countries**

Jinhua Pan^1†^ M.Sc., Ye Yao^1†^ Ph.D, Zhixi Liu^1†^ B.Med., Xia Meng^1^ Ph.D., John S Ji^2^ Ph.D., Yang Qiu^3^ Ph.D., Weidong Wang^1^ B.Med., Lina Zhang^1^ M.Sc., Weibing Wang^1, 4^ Ph.D., Haidong Kan^1*^ Ph.D.

^1^ School of Public Health, Key Lab of Public Health Safety of the Ministry of Education, Fudan University, Shanghai 200032, China;

^2^ Environmental Research Center, Duke Kunshan University, Kunshan, Jiangsu, China; Nicholas School of the Environment, Duke University, Durham, NC, USA.

^3^ Department of Environmental Sciences and Engineering, School of Architecture and Environmental Sciences, Sichuan University, Chengdu, China.

^4^ Shanghai Key Laboratory of Meteorology and Health, Shanghai, China

^†^Ms. Pan, Dr. Yao and Ms. Liu contributed equally to this work.

*** Corresponding Authors**: Dr. Haidong Kan, School of Public Health, Fudan University, Shanghai 200032, China, Email: [kanh@fudan.edu.cn](mailto:kanh@fudan.edu.cn)

**Supplemental Table 1**. Description of meteorological characteristics of 202 locations worldwide

|  | wind_speed[median(IQR)] | avg_temperature[median(IQR)] | relative_humidity[median(IQR)] | EDD[median(IQR)] |
| --- | --- | --- | --- | --- |
| Abruzzo | 2.10(2.10,2.10) | 8.98(8.98,8.98) | 83.89(83.89,83.89) | NA(NA,NA) |
| Aichi | 3.42(2.41,4.69) | 7.70(7.04,9.48) | 64.65(56.48,73.26) | NA(NA,NA) |
| Alabama | 2.47(1.97,2.88) | 16.79(14.07,19.52) | 80.43(64.69,87.01) | NA(NA,NA) |
| Alaska | 3.22(2.34,3.88) | -6.71(-10.46,-2.41) | 90.79(88.21,92.52) | NA(NA,NA) |
| Alberta | 3.82(2.82,4.63) | -5.50(-10.10,-1.84) | 72.12(65.64,76.96) | 597.64(505.11,935.58) |
| Anqing | 2.05(1.60,2.35) | 7.30(4.95,8.97) | 80.50(68.50,91.00) | 1156.41(801.29,1781.90) |
| Arizona | 2.88(2.33,3.56) | 10.44(9.45,12.16) | 59.64(45.60,69.19) | NA(NA,NA) |
| Arkansas | 1.96(1.32,2.61) | 13.73(11.18,15.39) | 83.05(70.21,88.40) | NA(NA,NA) |
| Australian Capital Territory | 1.99(1.76,2.65) | 16.51(15.46,17.69) | 70.31(64.95,76.37) | NA(NA,NA) |
| Baden-Wurttemberg | 4.21(3.52,5.64) | 6.04(4.47,8.46) | 77.79(70.28,80.65) | NA(NA,NA) |
| Bangbu | 2.20(1.80,3.00) | 5.80(3.87,8.20) | 79.00(72.00,87.50) | 1218.19(748.74,1537.69) |
| Basilicata | 7.19(4.97,9.17) | 9.42(7.02,11.02) | 69.77(63.38,77.87) | NA(NA,NA) |
| Bayern | 4.93(3.38,6.34) | 5.32(3.72,7.60) | 74.40(70.09,80.47) | NA(NA,NA) |
| Beijing | 1.50(1.20,2.30) | 0.90(-1.45,2.32) | 58.50(40.75,69.75) | 839.74(698.64,1072.64) |
| Berlin | 5.21(3.69,6.48) | 6.33(5.33,8.13) | 77.85(71.10,81.97) | 2987.37(2088.35,3886.38) |
| Bolzano | 1.68(1.36,1.89) | -0.68(-2.03,3.19) | 71.22(64.43,76.00) | NA(NA,NA) |
| Bozhou | 2.40(1.83,3.00) | 5.85(3.60,9.47) | 75.00(62.50,82.75) | 1113.30(683.02,1459.72) |
| British Columbia | 1.91(1.65,2.42) | 0.78(-0.78,2.58) | 74.06(68.08,77.88) | 545.79(423.93,788.06) |
| Buckinghamshire | 4.47(3.60,5.20) | 5.67(4.53,7.62) | 84.55(80.14,90.05) | 820.97(707.09,1328.10) |
| Calabria | 3.41(2.61,4.55) | 7.43(6.22,8.38) | 70.91(64.14,79.81) | NA(NA,NA) |
| California | 2.09(1.66,2.56) | 11.24(9.56,12.25) | 64.86(55.24,76.64) | 1972.76(1685.79,2162.90) |
| Cambridgeshire | 5.40(3.77,7.43) | 6.71(4.30,7.60) | 82.23(76.83,84.14) | 1458.34(915.58,1745.88) |
| Campania | 2.60(2.30,3.30) | 10.52(9.66,11.48) | 74.64(66.45,80.21) | NA(NA,NA) |
| Changde | 2.10(1.80,2.40) | 9.00(5.40,10.80) | 81.00(71.00,89.00) | 1310.22(825.75,1829.99) |
| Changsha | 2.10(1.20,3.60) | 8.05(5.95,10.55) | 83.00(69.50,95.00) | 1091.85(563.77,2159.19) |
| Chengdu | 1.30(1.10,1.50) | 8.65(7.45,9.90) | 79.00(73.75,82.25) | 1222.98(869.35,1620.93) |
| Chongqing | 0.90(0.60,1.30) | 10.45(9.40,11.70) | 82.00(73.00,85.50) | 1127.28(870.40,1414.22) |
| Colorado | 3.83(3.25,4.35) | 1.57(-1.06,3.45) | 61.65(48.47,67.23) | 2262.78(1976.83,2623.64) |
| Connecticut | 3.43(2.98,3.93) | 6.01(3.94,8.10) | 55.66(40.55,76.46) | NA(NA,NA) |
| Cumbria | 6.49(5.04,8.51) | 3.89(2.84,5.85) | 86.67(82.28,89.38) | 1173.89(959.54,1375.55) |
| Delaware | 3.62(3.25,3.94) | 10.34(9.48,12.05) | 95.03(94.49,97.51) | NA(NA,NA) |
| Doncaster | 4.49(3.35,7.02) | 6.42(5.21,8.40) | 76.26(70.89,79.03) | 6328.91(1513.13,6653.31) |
| Dongguan | 1.70(1.30,2.40) | 16.00(12.10,18.90) | 82.00(73.00,88.00) | 2588.26(1793.08,3267.86) |
| Dorset | 7.94(6.29,9.41) | 7.84(7.20,8.86) | 77.72(70.28,88.84) | 7005.46(6918.95,7055.56) |
| East Riding of Yorkshire | 5.15(3.91,6.77) | 6.02(4.55,7.72) | 79.33(77.58,82.85) | 1486.09(1045.18,3714.75) |
| East Sussex | 3.69(2.77,5.18) | 6.83(6.20,7.72) | 78.81(68.09,88.89) | 1620.86(1205.49,1864.45) |
| Emilia-Romagna | 1.59(1.32,2.18) | 8.63(7.45,9.69) | 76.06(68.49,81.57) | NA(NA,NA) |
| Enshi | 0.70(0.50,0.90) | 8.30(7.20,10.70) | 83.00(73.00,88.00) | 1177.53(928.79,1555.94) |
| Essex | 6.16(4.71,7.25) | 6.72(5.48,8.68) | 79.84(73.62,84.51) | 6995.63(6664.04,7088.70) |
| Ezhou | 1.90(1.50,3.00) | 7.50(5.30,9.80) | 80.00(69.00,89.00) | 987.19(753.76,1798.91) |
| Florida | 2.45(1.80,3.18) | 20.29(15.81,22.24) | 75.16(70.78,79.08) | 3100.78(2609.72,3693.70) |
| Foshan | 1.90(1.58,2.42) | 16.15(11.98,18.80) | 82.00(74.00,88.00) | 2425.24(1569.66,3043.49) |
| Friuli-Venezia Giulia | 1.97(1.59,2.81) | 8.71(7.64,10.09) | 74.88(67.44,82.03) | NA(NA,NA) |
| Fuyang | 2.40(1.80,3.38) | 6.10(4.22,9.73) | 78.50(68.25,84.75) | 1165.90(862.61,1456.11) |
| Fuzhou | 1.90(1.50,2.70) | 11.20(8.95,15.15) | 77.00(68.00,89.00) | 1778.71(966.44,2663.38) |
| Ganzhou | 1.30(1.10,1.60) | 11.30(8.50,16.40) | 75.00(67.00,86.00) | 2140.15(1049.97,2848.64) |
| Georgia | 1.80(1.54,2.37) | 16.05(11.30,19.24) | 76.38(63.44,82.26) | 2528.72(1681.37,2922.30) |
| Gloucestershire | 6.45(5.06,7.37) | 6.49(5.22,7.19) | 79.36(71.69,89.31) | 1608.02(932.65,1826.24) |
| Guangzhou | 1.90(1.58,2.42) | 16.15(11.98,18.80) | 82.00(74.00,88.00) | 2422.05(1515.04,3156.89) |
| Hamburg | 4.96(3.71,6.60) | 5.92(4.68,7.72) | 76.98(72.12,83.91) | 6284.36(1106.88,6403.36) |
| Hampshire | 5.87(4.95,6.76) | 7.25(5.57,8.72) | 77.56(71.77,84.67) | 1752.58(1289.42,6977.55) |
| Hangzhou | 2.15(1.50,2.62) | 8.20(6.68,10.80) | 76.00(61.00,91.00) | 1441.70(779.59,1755.28) |
| Harbin | 2.30(1.92,3.03) | -13.80(-16.98,-7.73) | 71.00(67.00,75.00) | 578.13(443.38,754.25) |
| Hawaii | 2.30(1.82,3.06) | 12.55(12.02,14.62) | 92.69(88.80,95.63) | NA(NA,NA) |
| Hefei | 2.25(1.67,3.12) | 5.85(3.97,8.10) | 84.50(73.75,92.25) | 1299.88(660.86,1685.46) |
| Hertfordshire | 0.00(0.00,0.00) | 6.24(5.05,8.03) | 82.00(78.94,86.79) | 1111.84(1033.48,1355.08) |
| Hessen | 5.15(4.07,6.23) | 7.75(6.14,9.10) | 75.11(69.05,78.35) | 1250.91(1079.32,2691.35) |
| Hokkaido | 3.68(2.96,4.20) | -1.94(-4.49,-0.62) | 73.88(71.06,80.79) | NA(NA,NA) |
| Huanggang | 1.85(1.50,2.70) | 7.10(5.27,9.72) | 80.50(68.50,90.00) | 1244.33(702.70,1761.71) |
| Huangshi | 1.20(1.00,1.52) | 7.00(4.62,8.45) | 78.50(66.75,83.25) | 1098.32(692.93,1847.24) |
| Huizhou | 1.90(1.40,2.40) | 16.60(13.30,19.90) | 77.00(67.00,84.00) | 2415.13(1712.57,3232.78) |
| Hyoto | 2.35(2.08,3.70) | 8.63(7.17,10.72) | 71.16(67.10,75.38) | 2479.84(2331.59,2628.10) |
| Idaho | 2.07(1.75,2.72) | 2.96(1.49,4.87) | 67.94(59.28,78.74) | NA(NA,NA) |
| Illinois | 3.49(2.68,4.71) | 5.58(2.72,7.29) | 80.32(65.65,89.82) | 1086.77(798.82,1652.28) |
| Indiana | 3.34(2.70,4.26) | 3.83(1.32,5.28) | 80.71(70.85,89.57) | 1069.52(741.71,1541.82) |
| Iowa | 4.20(3.25,5.34) | 3.79(1.06,6.66) | 80.80(72.64,89.67) | 1262.69(1002.03,1560.42) |
| Jingzhou | 1.60(1.10,2.15) | 7.40(5.95,10.40) | 82.00(78.50,90.00) | 1182.08(642.74,1726.49) |
| Jinhua | 1.55(1.10,1.98) | 9.05(7.03,12.97) | 76.50(60.00,90.00) | 1575.82(948.03,2197.41) |
| Jiujiang | 2.70(1.80,4.18) | 3.70(-0.35,8.15) | 91.50(70.25,97.00) | 1174.89(614.92,1824.89) |
| Kansas | 4.05(3.29,4.92) | 10.41(7.35,12.94) | 66.23(60.50,82.01) | NA(NA,NA) |
| Kent | 7.37(6.20,9.41) | 7.36(6.43,8.44) | 83.91(80.14,88.62) | 6890.26(6753.38,6997.89) |
| Kentucky | 2.62(2.05,3.18) | 9.25(4.35,11.52) | 80.21(59.14,87.11) | 1533.67(1012.31,1907.81) |
| Kirklees | 6.73(4.87,9.47) | 4.59(3.77,6.51) | 81.43(76.53,84.89) | 6432.67(1352.85,6761.65) |
| Lancashire | 6.29(4.25,8.22) | 7.21(6.26,7.92) | 76.24(67.81,84.38) | NA(NA,NA) |
| Lazio | 3.49(2.92,4.50) | 9.85(8.90,10.93) | 74.63(70.50,81.00) | NA(NA,NA) |
| Leeds | 5.70(4.77,7.61) | 4.80(3.89,6.64) | 82.77(76.50,84.63) | NA(NA,NA) |
| Leicestershire | 5.96(4.39,8.07) | 5.49(4.10,7.55) | 77.39(72.49,81.16) | NA(NA,NA) |
| Liguria | 2.91(2.47,3.54) | 9.25(8.66,10.16) | 76.18(61.79,84.66) | NA(NA,NA) |
| Lincolnshire | 7.03(5.60,8.83) | 6.43(4.71,7.57) | 80.27(76.12,82.86) | NA(NA,NA) |
| Lombardia | 1.93(1.65,2.46) | 7.85(6.85,8.94) | 71.19(59.77,81.67) | NA(NA,NA) |
| Loudi | 0.85(0.50,1.80) | 8.20(6.60,10.88) | 84.00(76.00,92.00) | 1306.90(768.44,1834.41) |
| Louisiana | 3.10(2.52,4.04) | 20.47(16.04,21.60) | 83.35(77.09,86.68) | 2886.36(2571.15,3024.84) |
| Lu'an | 1.70(1.30,1.95) | 7.10(4.20,9.85) | 80.00(64.50,90.50) | 1229.18(886.78,1621.82) |
| Luton | 5.41(4.34,6.56) | 5.33(4.43,6.96) | 78.24(72.49,84.87) | 7060.23(6827.13,7161.39) |
| Maine | 1.82(1.37,2.62) | -1.24(-5.07,1.16) | 88.55(83.07,92.10) | NA(NA,NA) |
| Manchester | 3.81(2.80,5.74) | 6.38(5.16,7.86) | 75.81(66.52,81.96) | 6583.90(6015.14,6891.00) |
| Manitoba | 3.59(3.02,4.58) | -5.57(-10.81,-2.93) | 74.01(70.75,78.78) | NA(NA,NA) |
| Marche | 3.63(2.92,4.84) | 9.78(8.38,11.00) | 58.48(53.49,67.61) | NA(NA,NA) |
| Maryland | 2.89(2.24,3.70) | 8.60(5.86,10.21) | 64.25(53.63,78.38) | 1229.25(713.60,1760.04) |
| Massachusetts | 3.72(2.77,4.27) | 5.47(3.43,8.50) | 62.27(48.50,79.31) | 1327.42(1040.37,1754.70) |
| Michigan | 3.36(2.83,4.48) | 1.06(-1.27,2.53) | 69.55(64.98,79.05) | NA(NA,NA) |
| Minnesota | 4.00(2.72,4.56) | 0.35(-2.87,3.52) | 77.17(68.81,81.94) | NA(NA,NA) |
| Mississippi | 2.70(1.82,3.03) | 16.84(13.51,18.51) | 83.09(73.70,86.86) | NA(NA,NA) |
| Missouri | 3.02(2.46,3.93) | 8.59(6.18,12.81) | 75.51(57.48,86.29) | NA(NA,NA) |
| Montana | 3.12(2.38,4.02) | -2.36(-4.43,1.64) | 81.24(71.45,86.88) | NA(NA,NA) |
| Nanchang | 1.30(1.05,1.95) | 9.30(7.60,11.65) | 79.00(69.00,91.00) | 1406.89(621.23,2071.12) |
| Nanjing | 2.25(1.83,2.95) | 7.55(5.00,9.10) | 75.50(67.25,93.00) | 1576.94(619.47,1829.90) |
| Nanyang | 2.30(1.90,2.80) | 6.10(3.70,8.00) | 78.00(62.00,87.00) | 1298.31(991.29,1502.28) |
| Nebraska | 4.00(3.55,5.02) | 4.49(2.20,7.09) | 72.76(63.62,86.83) | 1469.19(960.95,1885.28) |
| Nevada | 2.53(1.98,3.50) | 6.01(3.47,7.16) | 52.93(43.33,63.22) | 2170.12(1790.18,2302.53) |
| New Brunswick | 3.37(2.51,4.35) | -3.51(-5.63,-0.72) | 72.66(59.14,79.37) | NA(NA,NA) |
| New Hampshire | 1.67(1.42,2.65) | 3.41(1.58,5.89) | 71.11(56.06,84.24) | NA(NA,NA) |
| New Jersey | 2.64(1.58,3.75) | 8.05(4.73,9.50) | 49.18(42.04,70.73) | 1638.59(1137.99,2175.28) |
| New Mexico | 3.28(2.88,4.12) | 8.46(6.29,10.25) | 53.20(38.17,62.44) | NA(NA,NA) |
| New South Wales | 3.53(3.15,3.82) | 19.86(19.11,21.02) | 67.09(61.38,72.60) | 5113.32(4700.81,6373.41) |
| New York | 2.59(2.17,3.15) | 2.27(0.02,4.43) | 73.61(61.58,83.74) | 952.08(745.84,1435.07) |
| Newfoundland and Labrador | 5.36(3.72,6.38) | -2.91(-5.33,-1.26) | 81.02(73.17,86.94) | NA(NA,NA) |
| Niedersachsen | 5.02(3.71,6.46) | 7.10(5.71,8.61) | 77.45(70.45,81.12) | 971.49(851.30,1079.91) |
| Ningbo | 2.30(1.60,3.05) | 8.80(6.70,11.20) | 76.00(64.50,91.50) | 1622.64(835.75,2170.06) |
| Nordrhein-Westfalen | 5.90(4.66,7.42) | 6.88(5.35,8.59) | 76.27(72.06,83.70) | NA(NA,NA) |
| Norfolk | 6.29(5.04,7.48) | 6.31(5.04,7.74) | 81.28(75.28,83.05) | 1656.81(1266.55,2696.81) |
| North Carolina | 2.27(1.50,2.87) | 11.48(9.28,15.37) | 73.13(60.46,83.63) | 1820.10(1562.40,2636.80) |
| North Dakota | 4.78(3.65,6.21) | -1.91(-4.88,1.47) | 83.68(79.81,87.22) | NA(NA,NA) |
| North Lincolnshire | 6.06(4.32,8.19) | 7.52(6.41,9.35) | 76.65(67.68,80.09) | 1442.16(1150.41,2200.53) |
| North Somerset | 6.03(5.05,7.43) | 5.67(4.45,7.32) | 84.39(77.55,89.59) | 4786.91(1844.75,7095.88) |
| North Yorkshire | 5.35(3.86,7.25) | 6.05(4.63,7.41) | 78.22(73.87,81.40) | 1366.45(1032.51,1568.92) |
| Northern Territory | 2.66(2.30,2.93) | 28.86(27.74,29.02) | 64.52(62.14,67.80) | NA(NA,NA) |
| Northumberland | 4.28(3.25,5.55) | 5.19(3.69,6.32) | 82.48(79.50,84.55) | 6849.86(6522.10,7244.45) |
| Nottinghamshire | 4.47(3.34,5.79) | 5.86(4.51,7.75) | 77.75(72.06,81.65) | 1508.58(1020.79,6435.47) |
| Nova Scotia | 3.93(3.55,5.21) | 0.02(-1.55,1.84) | 73.58(62.66,85.73) | NA(NA,NA) |
| Ohio | 3.60(2.31,4.38) | 5.09(1.76,8.49) | 79.49(72.38,83.96) | NA(NA,NA) |
| Oklahoma | 3.64(2.77,4.93) | 11.69(9.25,16.26) | 71.35(63.32,87.36) | NA(NA,NA) |
| Ontario | 3.38(2.93,3.98) | -3.70(-7.72,-1.08) | 72.41(66.79,77.23) | 744.62(610.39,878.89) |
| Oregon | 1.68(1.41,1.96) | 4.84(3.23,6.57) | 73.31(70.82,79.93) | 1628.49(1231.74,1998.96) |
| Osaka | 2.10(1.78,2.95) | 9.24(8.16,10.82) | 59.96(53.56,66.27) | 2466.02(2466.02,2466.02) |
| Oxfordshire | 4.92(4.24,5.93) | 6.29(4.93,8.05) | 78.98(77.12,86.19) | 1323.10(805.79,6919.17) |
| Pennsylvania | 2.80(2.22,3.50) | 6.28(3.10,7.74) | 60.99(53.94,77.09) | 1281.06(906.83,1892.39) |
| Peterborough | 5.49(4.32,7.38) | 5.91(4.88,7.45) | 77.46(70.78,80.40) | 7056.60(6783.00,7134.23) |
| Piemonte | 1.37(1.09,1.96) | 5.45(4.47,7.44) | 65.00(53.85,80.47) | NA(NA,NA) |
| Pingdingshan | 1.90(1.50,2.70) | 4.50(3.00,7.60) | 76.00(66.00,88.00) | 1221.12(721.94,1350.63) |
| Plymouth | 5.58(4.12,7.68) | 7.79(7.00,8.90) | 82.33(68.98,91.25) | 6880.70(6677.63,7003.20) |
| Puglia | 3.68(2.77,5.30) | 10.32(9.38,12.08) | 73.29(70.11,76.79) | NA(NA,NA) |
| Putian | 3.80(2.75,5.10) | 13.20(12.25,16.10) | 73.00(64.00,84.00) | 2759.54(1591.22,3175.17) |
| Qingdao | 3.35(2.58,4.25) | 3.75(2.63,5.82) | 72.00(62.00,84.50) | 983.84(764.06,1143.71) |
| Quebec | 3.46(2.55,4.10) | -5.66(-8.99,-1.89) | 71.61(67.23,79.72) | NA(NA,NA) |
| Queensland | 3.97(3.31,4.40) | 26.56(25.80,27.04) | 69.14(62.51,74.72) | 5973.16(5290.81,6707.44) |
| Redcar and Cleveland | 7.43(5.24,8.97) | 6.04(4.70,7.80) | 79.00(70.69,82.23) | 4977.93(1131.79,6645.83) |
| Rheinland-Pfalz | 6.00(4.41,6.90) | 4.68(3.09,7.14) | 85.58(81.32,90.63) | 1511.80(1175.91,6140.07) |
| Rhode Island | 2.68(2.01,3.12) | 5.17(3.73,6.94) | 84.50(82.34,86.66) | NA(NA,NA) |
| Sardegna | 5.06(3.47,6.54) | 13.09(11.93,13.89) | 79.71(73.23,82.74) | NA(NA,NA) |
| Saskatchewan | 4.02(3.55,4.80) | -7.18(-11.80,-2.60) | 70.81(68.30,76.86) | NA(NA,NA) |
| Sefton | 5.62(3.90,6.55) | 7.37(6.30,8.99) | 76.99(69.96,81.87) | 6490.54(5096.50,6621.76) |
| Shanghai | 2.60(1.40,3.00) | 7.00(5.30,9.70) | 78.00(68.00,84.00) | 1608.41(1278.15,2058.72) |
| Shangqiu | 2.20(1.55,2.80) | 4.20(1.50,7.65) | 75.00(66.50,84.00) | 1076.30(884.61,1283.02) |
| Shangrao | 1.70(1.40,2.10) | 9.40(6.80,13.30) | 77.00(69.00,89.00) | 1455.75(1024.18,1951.59) |
| Shaoyang | 0.90(0.50,1.90) | 8.30(6.60,11.10) | 84.00(76.00,92.00) | 1678.41(960.71,2066.59) |
| Shenzhen | 1.60(1.30,1.80) | 17.50(15.70,20.20) | 76.00(68.00,82.00) | 2771.22(1937.14,3510.27) |
| Shiyan | 1.50(1.20,1.80) | 6.00(3.75,8.35) | 71.00(65.00,81.75) | 1614.44(1117.26,1721.02) |
| Shropshire | 5.27(4.22,8.01) | 6.09(4.06,7.52) | 79.47(76.45,81.63) | 1677.74(1127.02,6881.80) |
| Sicily | 3.14(2.67,3.82) | 12.94(11.95,13.74) | 77.12(72.77,80.07) | NA(NA,NA) |
| Solihull | 4.06(3.29,5.06) | 5.83(4.99,7.57) | 74.45(69.61,81.69) | 6704.53(6022.80,6895.34) |
| Somerset | 6.13(4.98,7.68) | 5.75(4.20,7.12) | 87.88(77.44,93.28) | 6955.42(6823.51,6990.03) |
| Soochow | 2.70(2.20,3.75) | 8.10(5.80,9.85) | 81.00(74.00,94.50) | 1293.46(820.12,1807.11) |
| South Australia | 4.58(4.03,5.32) | 18.18(17.43,20.09) | 59.91(51.27,64.52) | NA(NA,NA) |
| South Carolina | 2.25(1.71,2.80) | 14.81(11.97,18.47) | 74.34(70.19,79.77) | 2428.22(1553.97,2723.69) |
| South Dakota | 3.40(2.80,3.85) | 1.37(-1.72,5.61) | 82.50(68.97,87.43) | NA(NA,NA) |
| Staffordshire | 6.13(4.26,7.35) | 4.72(3.30,6.10) | 87.77(77.81,89.42) | 1477.36(881.53,6454.23) |
| Stockton-on-Tees | 6.96(4.98,8.46) | 7.18(6.03,8.45) | 73.80(67.05,75.92) | 6451.65(2007.26,7023.49) |
| Suffolk | 5.36(4.37,6.56) | 6.65(4.76,8.61) | 80.12(72.53,83.29) | 1572.18(1010.26,1784.75) |
| Suizhou | 1.50(1.10,1.90) | 7.70(4.20,10.30) | 72.00(60.00,84.00) | 1319.74(847.41,1653.44) |
| Surrey | 4.92(3.42,5.91) | 6.62(5.30,8.21) | 80.69(76.43,87.95) | 1535.94(948.04,3159.52) |
| Taizhou | 1.75(1.50,2.10) | 10.05(8.38,13.05) | 76.00(70.25,87.25) | 1883.16(1129.87,2469.33) |
| Tasmania | 5.02(3.41,5.78) | 12.01(9.98,13.36) | 80.17(77.72,83.57) | NA(NA,NA) |
| Tennessee | 2.98(1.96,3.44) | 11.55(8.50,13.87) | 81.64(65.97,88.55) | 1546.16(1290.00,2030.60) |
| Texas | 3.80(3.31,4.44) | 15.57(11.59,18.16) | 75.25(63.10,83.35) | 2676.24(1880.34,3012.55) |
| Tianjin | 2.00(1.45,2.70) | 1.60(-0.95,3.80) | 70.00(52.00,77.50) | 908.99(795.61,1018.75) |
| Tokyo | 2.52(2.17,3.00) | 8.70(7.57,10.47) | 51.21(45.50,63.29) | NA(NA,NA) |
| Toscana | 1.74(1.50,2.29) | 10.46(9.54,11.63) | 80.48(72.88,86.87) | NA(NA,NA) |
| Trento | 3.13(2.50,5.27) | -2.20(-4.80,1.86) | 63.29(50.39,77.35) | NA(NA,NA) |
| Utah | 1.94(1.61,2.99) | 5.42(2.59,6.74) | 52.87(43.36,77.84) | NA(NA,NA) |
| Valle d'Aosta | 6.77(4.50,8.33) | -3.01(-4.31,1.93) | 62.10(56.15,72.87) | NA(NA,NA) |
| Veneto | 1.93(1.65,2.35) | 7.68(6.86,8.39) | 78.74(72.32,86.67) | NA(NA,NA) |
| Vermont | 2.86(1.87,3.87) | 1.60(-0.88,4.15) | 71.90(55.34,77.63) | NA(NA,NA) |
| Victoria | 4.04(3.47,4.41) | 15.40(14.49,18.45) | 68.12(62.94,71.09) | 3720.22(3444.95,4277.59) |
| Virginia | 2.47(1.85,3.65) | 9.47(6.12,10.76) | 70.75(55.65,78.55) | 1690.04(1177.96,2365.54) |
| Warwickshire | 4.26(3.36,5.20) | 6.35(5.05,8.06) | 79.89(75.45,83.95) | 1509.48(768.16,6749.06) |
| Washington | 1.63(0.93,1.94) | 4.30(2.89,5.29) | 83.04(77.32,88.01) | 900.07(754.34,1033.83) |
| Wenzhou | 1.70(1.50,2.18) | 11.25(9.20,13.72) | 74.00(66.75,87.50) | 1853.15(1143.23,2271.62) |
| West Sussex | 5.89(4.85,7.35) | 7.30(6.32,8.61) | 78.17(73.74,90.85) | 1759.87(1235.67,4176.15) |
| West Virginia | 1.68(1.18,2.55) | 10.08(8.01,12.87) | 75.01(60.20,82.50) | NA(NA,NA) |
| Western Australia | 4.21(3.63,4.69) | 24.27(23.69,25.18) | 58.01(54.86,62.41) | NA(NA,NA) |
| Wiltshire | 5.38(4.42,6.16) | 6.16(4.84,7.82) | 82.70(75.72,88.89) | 1847.14(1459.82,5721.60) |
| Wisconsin | 3.07(2.10,3.89) | 0.13(-2.43,3.04) | 69.90(63.22,82.69) | NA(NA,NA) |
| Worcestershire | 4.31(3.09,5.83) | 6.55(5.16,8.87) | 79.21(75.54,82.59) | 1699.17(1133.70,6766.50) |
| Wuhan | 1.35(0.88,1.83) | 5.60(3.68,8.15) | 85.50(79.00,90.00) | 856.23(564.83,1516.12) |
| Wyoming | 3.84(3.22,4.45) | -0.15(-1.89,1.46) | 72.01(66.70,78.56) | NA(NA,NA) |
| Xi'an | 1.85(1.20,2.25) | 4.85(3.40,7.62) | 58.00(45.75,67.25) | 1529.34(1125.55,1734.30) |
| Xiangyang | 1.85(1.50,2.40) | 6.30(4.75,8.27) | 82.00(75.75,94.00) | 1424.51(945.36,1630.43) |
| Xianning | 2.15(1.72,2.70) | 8.55(5.80,10.58) | 78.50(65.00,88.25) | 1187.39(676.30,1946.89) |
| Xiaogan | 1.35(0.80,1.78) | 6.60(4.58,9.97) | 83.00(77.00,88.00) | 1142.23(729.09,1651.94) |
| Xinyang | 1.90(1.52,2.58) | 7.75(5.05,10.60) | 76.00(60.00,86.00) | 1182.45(632.89,1489.11) |
| Xinyu | 1.85(1.40,2.40) | 9.20(7.55,13.08) | 77.00(69.25,90.25) | 1430.43(715.00,2342.81) |
| Xuzhou | 1.60(1.25,2.15) | 5.50(2.80,9.35) | 70.00(61.00,80.00) | 1189.97(743.99,1499.36) |
| Yichang | 1.90(1.63,2.30) | 8.00(5.70,9.95) | 77.00(70.25,89.50) | 1519.86(782.15,1775.67) |
| Yichun | 1.90(1.40,2.40) | 9.20(7.50,13.30) | 77.00(69.00,88.00) | 1396.29(728.19,2229.48) |
| Yiyang | 1.30(1.00,1.80) | 9.00(6.60,10.80) | 89.00(75.00,97.00) | 1179.01(706.59,1867.94) |
| Yueyang | 2.00(1.42,2.77) | 9.10(6.40,11.02) | 82.50(69.25,94.00) | 1100.62(633.81,2105.42) |
| Zhengzhou | 1.50(1.20,2.05) | 4.60(3.25,8.05) | 71.00(56.50,80.00) | 985.41(668.65,1171.75) |
| Zhongshan | 1.60(1.33,1.80) | 17.65(15.10,20.28) | 76.50(68.00,82.75) | 2317.42(1804.91,3034.42) |
| Zhoukou | 1.90(1.42,2.38) | 6.45(3.50,9.25) | 79.50(59.50,86.75) | 1142.12(652.85,1246.09) |
| Zhuhai | 1.60(1.28,1.80) | 17.65(15.50,20.22) | 76.50(67.75,82.25) | 2838.00(1975.12,3428.36) |
| Zhumadian | 2.30(1.70,2.80) | 5.60(3.60,8.30) | 67.00(58.00,79.00) | 1367.97(967.27,1418.87) |


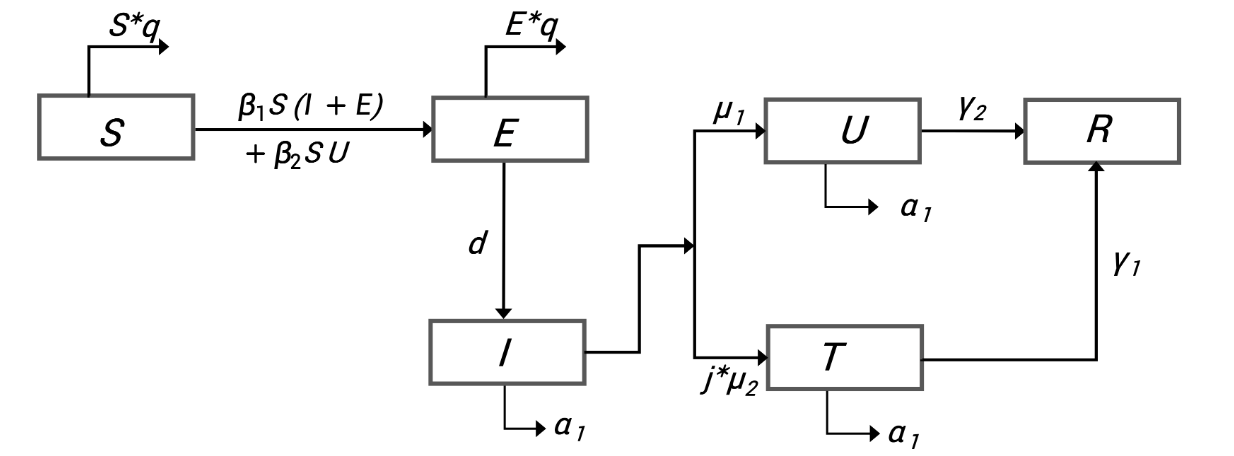


**Figure S1. Flow diagram for the model**

β_1_ represents the probability of transmission following a contact between infectious and exposed cases and susceptible individuals, β_2_ represents the probability of transmission following a contact between subclinical cases and susceptible individuals, q is the quarantine rate, j is the detection rate, α_1_ is the death rate, γ_1_ and d are progression rate of cases from confirmed to recovery and exposed to infection, respectively. μ_1_ and μ_2_ are ratios of subclinical and confirmed cases.
